# Cross-Sectional Study on the Prevalence and Factors Influencing Occurrence of Tick-Borne Encephalitis in Horses in Lithuania

**DOI:** 10.3390/pathogens10020140

**Published:** 2021-01-31

**Authors:** Arnoldas Pautienius, Austeja Armonaite, Evelina Simkute, Ruta Zagrabskaite, Jurate Buitkuviene, Russell Alpizar-Jara, Juozas Grigas, Indre Zakiene, Dainius Zienius, Algirdas Salomskas, Arunas Stankevicius

**Affiliations:** 1 Virology Laboratory, Institute of Microbiology and Virology, Faculty of Veterinary Medicine, Lithuanian University of Health Sciences, Tilzes str. 18, LT-47181 Kaunas, Lithuania; juozas.grigas@lsmuni.lt; 2 Laboratory of Immunology, Department of Anatomy and Physiology, Faculty of Veterinary Medicine, Lithuanian University of Health Sciences, Tilzes str. 18, LT-47181 Kaunas, Lithuania; austeja.armonaite@lsmuni.lt (A.A.); evelina.simkute@lsmuni.lt (E.S.); indre.zakiene@lsmuni.lt (I.Z.); arunas.stankevicius@lsmuni.lt (A.S.); 3 National Food and Veterinary Risk Assessment Institute, J. Kairiukscio Str. 10, LT-08409 Vilnius, Lithuania; ruta.zagrabskaite@nmvrvi.lt (R.Z.); jurate.buitkuviene@nmvrvi.lt (J.B.); 4 Research Center in Mathematics and Applications (CIMA-UE), Institute for Advanced Studies and Research, Department of Mathematics, School of Science and Technology, University of Évora, Rua Romão Ramalho 59, 7000-671 Évora, Portugal; alpizar@uevora.pt; 5 Department of Veterinary Pathobiology, Faculty of Veterinary Medicine Lithuanian University of Health Sciences, Tilzes str. 18, LT-47181 Kaunas, Lithuania; dainius.zienius@lsmuni.lt (D.Z.); algirdas.salomskas@lsmuni.lt (A.S.)

**Keywords:** TBE, TBEV, tick-borne encephalitis, TBE seroprevalence

## Abstract

Various animal species have been evaluated in depth for their potential as Tick-borne encephalitis virus (TBEV) sentinel species, although evidence for equine capacity is incomplete. Therefore, a comprehensive cross-sectional stratified serosurvey and PCR analysis of selected horses (*n* = 301) were performed in TBEV endemic localities in Lithuania. Attached and moving ticks (*n* = 241) have been collected from aforementioned hosts to evaluate natural infectivity of TBEV vectors (*Ixodes spp.*) in the recreational environments surrounding equestrian centers. All samples were screened for TBEV IgG and positive samples were confirmed by virus neutralization test (VNT). 113 (37.5%) horses from all counties of Lithuania tested positive for TBEV IgG, revealing age and sex indifferent results of equine seroprevalence that were significantly dependent on pedigree: horses of mixed breed were more susceptible to infection possibly due to their management practices. TBEV prevalence in equine species corresponded to TBEV-confirmed human cases in the precedent year. As much as 3.9% of horses were viraemic with TBEV-RNA with subsequent confirmation of TBEV European subtype. 4/38 of tested tick pools were positive for TBEV-RNA (Minimal infectious rate 1.2%). Several unknown microfoci were revealed during the study indicating areas of extreme risk close to popular human entertainment sites. The study provides important evidence in favor of horses’ usage as sentinel species, as equines could provide more detailed epidemiological mapping of TBEV, as well as more efficient collection of ticks for surveillance studies.

## 1. Introduction

Tick-borne encephalitis (TBE) is the most important tick-borne viral zoonotic disease in Europe caused by a bite of TBE virus (Flavivirus, Flaviviridae) infected *Ixodes* spp. [1]. In Lithuania TBE incidence rates have been increasing by 8.5% per year for the 45-year period from 1970 to 2014 [2]. Moreover, the country has the highest incidence rate of the disease in Europe since 2013 [3].

Tick-borne encephalitis is endemic throughout Lithuania [4]. The number of autochthonous confirmed clinical TBE cases with known site of exposure define a risk level for TBE in the region. Since the actual population at risk can deviate greatly from the number of inhabitants in an area due to the focal distribution of TBE, risk estimates based on incidence have limitations, especially if large geographic areas are chosen [5]. In addition, socioeconomic factors, preventive measures such as high vaccine coverage and recreational usage of a natural area can significantly alter results of disease monitoring, necessitating alternative methods to define the degree of risk and endemicity.

Wild and domestic animals have already raised an interest as surrogate markers of natural Tick-borne encephalitis virus (TBEV) prevalence [6,7,8]. Significant correlation between seroprevalence in small mammals, dogs, bovids, cervids and TBE incidence in humans was confirmed in known endemic areas [9,10,11,12,13], as well as the capacity of these sentinel species to uncover presently unknown TBEV foci, whereas insights to equine contribution to wild TBEV dynamics are lacking [6].

Clinical TBE cases in veterinary medicine are rare but can manifest with varying degree of non-specific neurological symptoms. In horses, reduced general condition, behavioral changes, ataxia and paralysis of neck and shoulder muscles have been described [14]. Laboratory diagnosis is essential for TBE confirmation, such as detection of TBEV IgM and/or TBEV IgG antibodies in serum or cerebrospinal fluid by immunoassays. Due to well known serological cross-reactivity with other flaviviruses, case validation requires subsequent confirmation of all enzyme-linked immunosorbent assay (ELISA) positive results by virus neutralization test (VNT) or PCR [14].

Horses have scarcely been investigated as potential TBEV sentinel species and only a few articles describe the epidemiological role of equines for TBEV circulation in nature [14,15,16]. Therefore, to evaluate equine contribution to TBEV and sentinel-species capacity we report a cross-sectional study performed on the prevalence and factors influencing occurrence of tick-borne encephalitis in horses in Lithuania.

## 2. Materials and Methods

### 2.1. Sample Collection and Sampling Sites

In order to accurately represent a cross-sectional study design that analyzes data collected at one given point in time [17], equine blood samples (*n* = 301) were collected in one month (May 2019), at the peak of first tick questing period. Stables were added to the study if they adhered to the inclusion criteria: sampled horses were turned out in pastures every day, had a well defined territorial radius and were not vaccinated for any flaviviruses.

After applying stratified random sample approach, 32 equestrian centers were examined (Table 1). Each stratum of equine blood samples in our study was estimated to represent equine population in each of the counties of Lithuania with a 5% margin of error (CI 95%) based on the national database provided by National Land Service under the Ministry of Agriculture of the Republic of Lithuania. To assess factors of influence to occurrence of the TBE, detailed information was collected regarding stable-level risk factors such as herd size, their ration composition, daily pasture time and animal-level risk factors: age, breed, gender of the horses, prior travel and health records. 

Blood was drawn with regard to animal welfare regulations [18], then serum samples of 3–4 mL in volume were separated by double centrifugation process, transferred to 2 mL tubes and stored at −20 °C until further use.

In addition, attached or moving ticks (*n* = 241) were collected from aforementioned hosts where possible. Pools were formed depending on the number of ticks collected from a given location, 5–10 nymphs or 2–5 adult ticks per pool. The ticks were dissected and homogenized in phosphate-buffered saline (PBS; 1×, pH 7.2; Gibco, Grand Island, NY, USA), inserted into liquid nitrogen and then ground into a fine powder in a mortar. Each homogenized suspension was centrifuged, supernatant was collected and stored at −20 °C. 

### 2.2. Data of Human TBEV Cases

Data concerning confirmed human TBEV cases in different counties of Lithuania were obtained from the Centre for Communicable Diseases and AIDS of Lithuania and population data for the calculation of TBE seroprevalence were obtained from the Lithuanian Department of Statistics.

### 2.3. ELISA and Virus Neutralization Test

The serum samples were tested by ELISA for TBEV IgG using the EIA TBEV IgG kit (TestLine Clinical Diagnostics, Brno-Královo Pole, Czech Republic) following manufacturer’s protocol. The results were calculated as the negative control/sample ratio, and a < 150% was used as a cutoff value for negative samples, 200%> for positive samples. All serum samples were retested by VNT for case confirmation and exclusion of serological cross-reactivities with other flaviviruses. The TBEV-specific neutralizing antibodies were determined using gold standard in-house neutralization assay [19]. Prior to testing, horse serum samples were complement inactivated and diluted starting from 1/5 to 1/320 in Minimum Essential Medium (MEM) (Gibco, Grand Island, NY, USA). The serum dilutions were incubated with 100 TCID_50_ of TBEV strain obtained after 10 passages of cultivation on Vero cell culture (ATCC^®^ CCL-81^™^, Manassas, VA, USA). Cells were assessed for the presence of cytopathogenic effects at 3, 5 and 7 days post-infection (p. i.). TBEV reciprocal titre of ≥1/20 was considered as positive in VNT.

Due to discrepancy of positive samples yielded after serology and VNT, we have raised a concern of possible concomitant circulation of multiple flaviviruses in Lithuania and performed an additional ELISA test for West Nile virus IgG using WN Competition Multi-species Ig kit (ID Screen, ID.vet, Grabels, France).

### 2.4. TBE Virus Detection and Viral Load Quantification

Total RNA was extracted from 300 µL serum and tick suspension samples using the GeneJET RNA Purification Kit (Thermo Scientific, Waltham, MA, USA) according to the manufacturer’s instructions. Samples were screened by conventional and real time reverse transcription PCR for the presence of specific TBEV RNA using primer sets described previously [20,21]. Reaction mix SuperScript™ III One-Step RT-PCR System with Platinum™ *Taq* DNA Polymerase (Thermo Scientific, Waltham, MA, USA) was utilized for real time PCR and DreamTaq Green PCR Master Mix (2×) (Thermo Scientific, Waltham, MA, USA) for conventional PCR. PCR reactions were carried out in triplicates.

We modified viral quantification assay as it was previously described [20]. Briefly, a synthetic fragment corresponding to the amplified region of the TBEV was cloned into the pJET1.2 vector using the CloneJET PCR Cloning Kit (Thermo Scientific, Waltham, MA, USA) and Transform-Aid Bacterial Transformation Kit (Thermo Scientific, Waltham, MA, USA) according to the manufacturer’s instructions. Following transformation of *E. coli* cells, plasmid DNA extraction and purification was performed using the GeneJET Plasmid Miniprep Kit (Thermo Scientific, Waltham, MA, USA) according to supplier’s protocol. Standard curves were generated after 10-fold dilutions of stock DNA (supplementary material Figure S1). Reactions were carried out in triplicates.

Quality of RNA extraction of tick samples was assessed using RT-qPCR targeting 16s rRNA of *I.ricinus* as described elsewhere [20].

### 2.5. Virus Isolation

Virus was isolated in Vero (ATCC^®^ CCL-81^™^, Manassas, VA, USA) cell line and serial passages were performed to assess its viability to infect cells and obtain sufficient number of viral copies necessary for sequencing.

Samples were passed through a 0.22-μm pore size microfilter (Techno Plastic Products AG, Trasadingen, Switzerland) for purification. Cells were then inoculated in 25 cm^2^ tissue culture flasks (TPP Techno Plastic Products AG, Trasadingen, Switzerland) for 1h at 37 °C. Negative controls were inoculated with MEM and 100 U mL^−1^ penicillin and 100 μg L^−1^ streptomycin only. The inoculate was removed and 10 mL of Minimum Essential Medium with additional 10% heat-inactivated fetal bovine serum (FBS; Gibco, Grand Island, NY, USA) was added. Cells were incubated at 37 °C in 5% CO_2_ and air mixture and was examined for the occurrence of cytopathic effect through 5 serial passages which were performed in triplicate set frame including triplicate of positive and negative controls for each round of analysis.

### 2.6. Sequencing and Phylogenetic Analysis

For further genetic characterization and positive sample confirmation partial genome sequencing was performed. Multiple alignment of all sequences was created using ClustalW software (Clustal, Dublin, Ireland) in MEGA X package. The neighbor-joining method was used for phylogenetic tree construction with 1000 bootstrapping replicates. Sequences of different TBEV strains and closely related flaviviruses chosen from the NCBI GenBank database were used for phylogenetic comparisons.

### 2.7. Statistical Analysis

Confidence intervals of seroprevalence were based on the exact binomial method. Binary logistic regression analysis was used to test the significance of the differences and odds ratio (OR), regression coefficient (β), and standard error (SE) in age-, sex- and breed-specific antibody prevalence. Chi-square test was used to calculate associations between pasture time and seropositivity. Pearson correlation was used to evaluate relation of human and horse TBEV cases. The minimal infection rate (MIR) was calculated as the ratio of the number of positive pools to the total number of ticks tested. MIR = number of positive pools/total number of ticks tested × 100%. Results with *p* value were regarded as significant. All statistical analysis and mapping was performed using the programming language R-project (4.03).

## 3. Results

### 3.1. ELISA and Neutralization Assay

**Figure 1:**
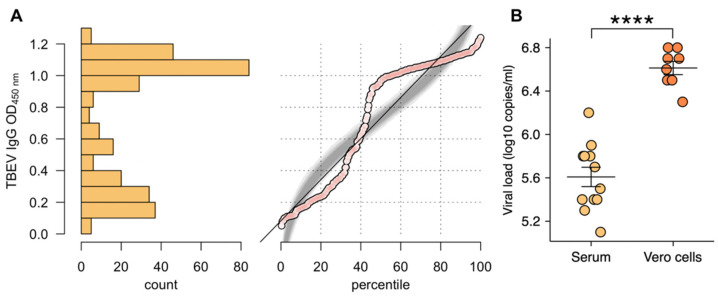
(**A**) Normal quantile plot of the distribution of optical density (OD) 450 nm values in Tick-borne encephalitis virus (TBEV) immunoglobulin G (IgG) enzyme-linked immunosorbent assay (ELISA) with sera collected from horses. Percentile plot with simulation (grey) of a normal distribution with the same mean and standard deviation as for the data collected; (**B**) Difference in TBE viral loads between cultured medium and serum-originated samples. (**** *p* < 0.0001).

Based on ELISA assay, 124 (41.2%), 5 (1.7%) and 172 (57.1%) serum samples were considered positive, borderline and negative for the presence of TBEV-specific antibodies, respectively. Thus, optical density plot showed clear bimodal distribution (Figure 1A). All samples were retested by virus neutralization assay. ELISA negative samples were consistent with VNT; however, only 109 and 4 samples were confirmed as positive in ELISA-positive and ELISA-borderline groups, respectively. Therefore, for further analysis, the overall number of TBE seropositive samples was adjusted according to VNT results at 113 (37.5%; 95% CI 32.2–43.1).

The study comprised 145 (48.2%) mares, 20 (6.6%) stallions and 136 (45.2%) geldings. No statistically significant associations were found between ration composition, herd size, age, gender and seropositivity.

Horses investigated in our study consisted of 26 breeds, as well as mixed breed individuals that represented almost a third of all tested equids (*n* = 85; 28.2%). Binary logistic regression model revealed significant association between the pedigree and serological results: seroprevalence of 31.0% (95% CI 24.9–37.6) and 53.4% (95% CI 42.41–64.3) of horses with known origin and mix-breeds, respectively (*p* < 0.03; OR 2.3; CI 95% 1.3–4.0; β 0.8; SE 0.3). In addition, we have found a significant relation between daily time spent in the pastures and seropositivity as horses that spent >8 h in the field had more than 2 times higher seroprevalence (68.0%; CI 95% 59.8–75.4) than horses that spent <8 h daily (31.7%; CI 95% 18.0–48.0). Notwithstanding the obvious difference, endmost result should be assessed ambiguously as the data was collected through a questionnaire survey of animal owners which may be affected by undefined bias.

Concerning equine sera tested with ELISA for detection of West Nile virus antibodies, we have found that some horses showed WNV seropositivity and negative TBEV VNT with 100 TCID_50_ of TBEV strain (data not shown).

Analysis of horse medical records showed no association with TBEV seropositivity. 11 horses demonstrated certain degree of atypical behavior or balance disturbances that we attributed to underdiagnosed cases of non-infectious origin, even though five of them were positive for TBEV neutralizing antibodies. Travel history of 17 horses included neighboring countries, although most of these equines did not travel outside Lithuania in the past 2 years at the time of sampling.

### 3.2. Virus Detection and Isolation

Due to a short time viremia, after which flaviviruses are cleared from serum, direct virus detection is limited in large herbivores and to our best knowledge we are first to report successful PCR and sequencing results in horses. As many as 12 (3.9%; 95% CI 2.3–6.8) of all tested equine serum samples were confirmed positive for the TBE virus. In addition, 4/38 of tested tick pools were positive for TBEV-RNA (MIR 1.2%) All positive tick samples were in adult tick stage of *I. ricinus* obtained from the same stable.

Presence of infective virus in serum samples and tick homogenates was confirmed by PCR analysis of the Vero cell culture supernatants collected at 5–6 days p.i. in all tested tick samples and 8 horse serum samples. Load of virus particles was assessed at different time points. Titres shown in this paper were assessed after the fourth passage at the load peak (Figure 1B)

### 3.3. Sequencing

**Figure 2:**
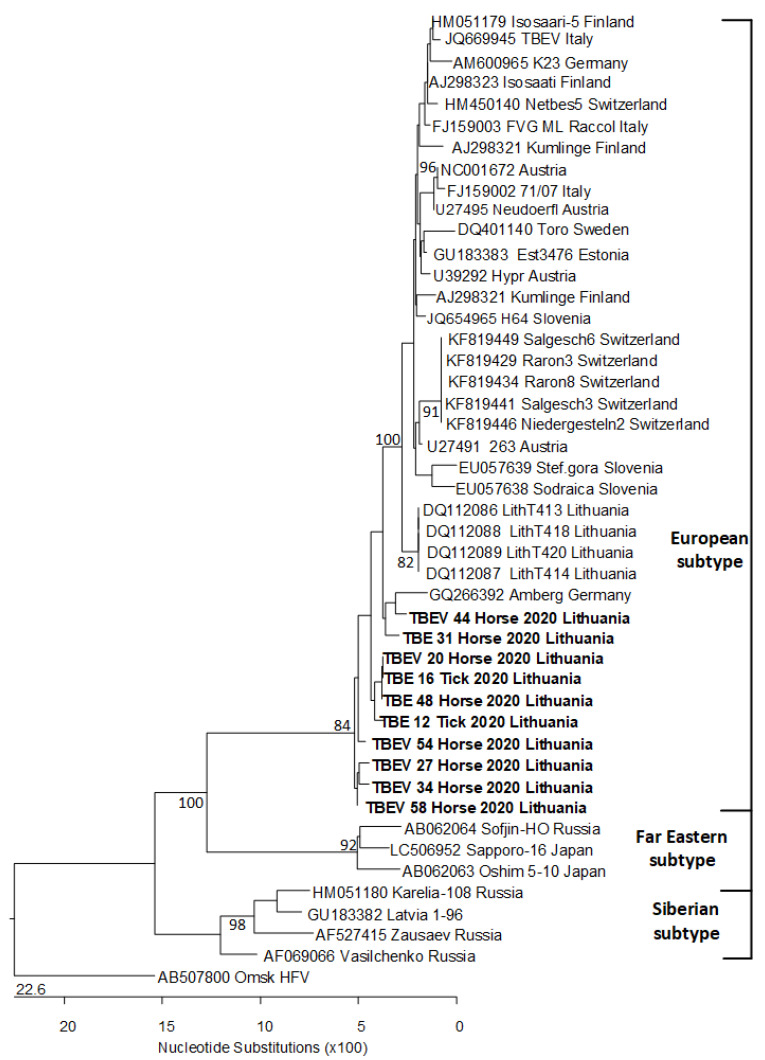
Phylogenetic tree of the TBEV sequences based on NS5 gene. The Omsk hemorrhagic fever virus was used as an outgroup. The sequences obtained in our study are shown in bold. Sequence data have been submitted to GenBank database under Accession Numbers MT981174-MT981178 and MW187721-MW187725.

Successful virus isolation was confirmed by partial genome sequencing based on NS5 fragment of TBEV isolates retrieved from cell cultures (Figure 2). Sequence data have been submitted to GenBank database under Accession Numbers MT981174-MT981178 and MW187721-MW187725. BLASTN search (NCBI Genbank) revealed that all DNA sequences of equine serum in this study were 93.0–97.1% similar to the reference strain of the European subtype (Neudörfl; Genbank U27495). In contrast, the similarity with the reference strains of the Far Eastern subtype (Sofjin; AB062064) and the Siberian subtype (Vasilchenko; AF069066) were 78.3 and 84.4%, respectively. TBEV sequences obtained from ticks in the present study shared a high degree of similarity (99.6%) to European subtype.

### 3.4. Spatial Distribution and of TBEV Specific Antibodies

**Figure 3:**
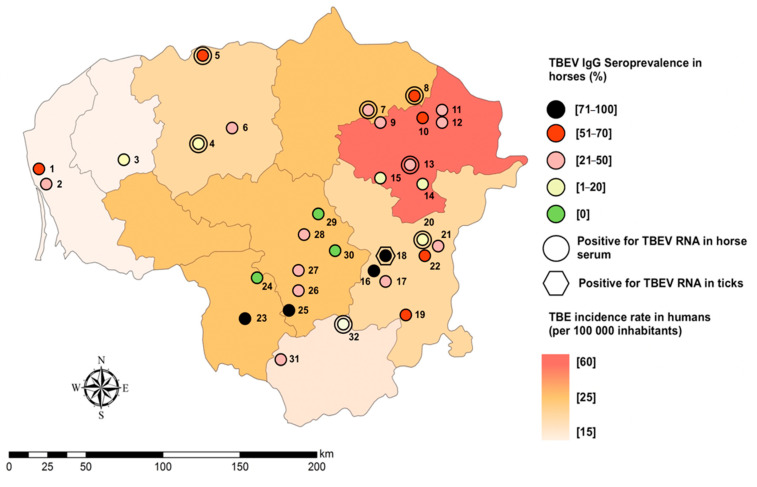
Spatial distribution of TBEV RNA and TBEV-neutralizing IgG seroprevalence at the county level in Lithuania. The colour grades represent the incidence rate in human TBE infection in 2019. Locations of sample acquisition are identified in numbers that are represented in Table 1. For interpretation of the references to colour in this figure legend, the reader is referred to the web version of this article.

Spatial pattern corresponding to the sampling locations is shown in Figure 3. To ensure anonymization and personal data protection, the geographic coordinates reflecting the exact location of the stables were randomly shifted to one of the cardinal directions in the range of 10 to 50 km without overstepping the stratum of sampling.

TBEV neutralizing antibodies were detected in 29 (90.6%, 95% CI 74.9–98.0) of all tested stables covering all counties involved in the study (Table 1).

Spatial analysis showed statistically significant differences in average seroprevalence amongst counties of the country. Highest seroprevalence rates were observed in east part of Lithuania with overall highest seroprevalence in Vilnius (50.6%; CI 95% 39.6–61.1) county (*p* < 0.04; OR 3.3; CI 95% 1.0–10.7; β 1.2; SE 0.6).

In the study year, insignificant positive correlation between TBEV seropositivity in tested horses and TBE-incidence rate in humans in given administrative unit was observed (r = 0.29). In contrast, strong positive correlation between these variables were detected with human TBE incidence of 2018 (r = 0.76; *p* < 0.05).

## 4. Discussion

Our study provides a first comprehensive investigation into prevalence of TBEV in equines in the northern Europe. We have revealed significantly higher seroprevalence of TBEV in horses (37.5% (95% CI 32.2–43.1) than previously recorded in Lithuania where in all domestic animal species tested TBE seropositivity of only 8.6% in 2003 and 1.7% in 2005 was found with considerable regional differences. Immense differences of results may be due to the strict inclusion criteria of sampling that allowed only horses with plausible exposure to TBEV infection to be examined e.g., sport horses that are ridden indoors were not included in the study. Interestingly, this upsurge of TBEV seroprevalence corresponds to the pattern observed in humans where a joinpoint analysis revealed 7.4% annual increase of TBEV incidence rate in 2005–2014 [2] in spite of characteristic fluctuations of TBE cases observed between tick questing seasons [22,23,24].

Contrary to the tendency observed by Rushton et al. [15], our study did not find significant associations between sex of the horse and seropositivity. This result may be influenced by different management conditions imposed on stallions in Austria [15], whereas in our study, stallions were mostly kept in comparable environments to those of mares. In addition to this, high tick density and infectivity in Lithuania may be underlying factor for non-differentiating results in both sexes.

In our study, age of the horses was an insignificant factor to TBEV infection unlike in equines in Austria where younger animals seemed to be more prone to infection [15] and previous studies of cattle, where animals up to 3 years old had significantly lower seroprevalence [25].

After having tested foals, we have found agreeing evidence that maternal immunity could be passed via colostrum, as most foals under 7 months old were seropositive with considerably high antibody values. Yearlings showed more varying results as an indication to fading antibodies of passive transfer.

One of the TBEV prevalence defining factor in our study was breed and more specifically, pedigree. As much as it can be tempting to rely on genetics, we think that this connection is purely based on the difference in management and purpose of mixed and purebred horses. Horses without defined pedigree are more likely to be used for long hikes in nature, as well as leisure and tourism. In addition, they were kept in pastures for significantly longer periods of time; therefore, they had higher probability of contracting a tick-borne infection.

Virus detection and sequencing processes revealed that as much as 3.9% (95% CI 2.3–6.8) of tested horses were positive for TBEV-RNA with subsequent confirmation of TBEV European subtype. To our best knowledge, the only study reporting sole sample potentially positive by PCR was performed in Germany. However, their sequencing or cultivation attempt was not successful [14]. Interestingly, the sequences we obtained did not cluster with other Lithuanian sequences. This can be explained by a lack of NS5 viral genome fragment-targeted or whole-genome sequences obtained in given territories as most of studies confirm specific product based on E or NS3 encoding fragments. Thus, further studies on whole genomic sequencing are necessary to get deeper insight on regional genetic diversity and lineages of TBEV strains.

Field collected ticks are known to be time and labor consuming indicators of infection risk [26,27], but it seems that infectivity of ticks is relatively higher, if samples are collected in the vicinity of domestic animals [14]. Our study revealed a relatively high prevalence of TBEV in ticks compared to a nationwide study conducted in Lithuania in 2017–2019 where MIR in field collected ticks was only 0.4% [28]. Almost 3-fold higher minimal infectious rate in ticks is most likely due to sampling properties: in our study ticks were collected from animals where favorable feeding conditions were guaranteed. The latter factor is associated with changes in dynamics of virus reproduction. It has been experimentally proven that intensive viral replication commences during feeding of ticks resulting in 500 times higher viral load in 15h period while in unfed ticks TBEV concentration remains stable [29]. In contrast, aforementioned study was based on sampling of questing ticks where some of the ticks potentially had low concentration of TBEV which therefore was below detection threshold. In addition, infected ticks are significantly more active and aggressive hence, there is a potentially higher probability of TBEV being found in the host rather than among questing ticks [29].

Important findings in this study seem to be the absolute seroprevalence and high values of TBEV IgG observed in equine sera obtained from several locations indicating a recent infection with TBEV from previously unknown microfoci close to these equestrian centers. In addition, ticks from one of these stables were found to have an infective TBEV confirmed by PCR and virus isolation. Previous studies have proven that sera of game animals is a reliable tool to reveal natural TBEV circulation [30] and we suggest that equine sera too is an important surrogate marker of areas of extreme risk.

Equine sentinel-species capacity is hard to evaluate. Our study has found significant correlation between equine TBEV seropositivity and human TBE cases for precedent year as equine individuals seem to respond accurately to the grade of infectivity in the geographical areas. On the other hand, seropositive results of horses failed to correspond to human incidence rates on the year of study (2019). We believe these results were biased due to relatively long period of time required for IgG production upon a new TBEV infection and population data discrepancy, as equine samples were collected in May and compared to human incidence rate of complete year in December. Cross-sectional design and population data of large geodemographic areas were important limitations of this study that precluded us from more complete evaluation of TBEV dynamics in horses, although several important aspects for TBEV sentinel species seem to be applicable to equines. Horses are capable to mount their Ab levels after contact with TBEV that could be detectable 9–19 months post-infection [14,31,32]. Interpretation of seroprevalence on the level of the stables could permit mapping of TBEV foci on smaller than district level as high Ab values of positive horses at the equestrian centers indicate recent TBEV exposure. In addition, multiple countries have currently established routine monitoring programs for equine infectious anemia therefore, large sample sizes are readily available. In addition to that, increased MIR of ticks collected from horses roaming large, well defined areas signifies an alternative to labor and time consuming field collection of ticks.

Interestingly, several equine serum samples appeared seropositive for WNV and demonstrated cytopathic effects after VNT with TBEV, indicating first serological evidence of WNV in Lithuania. These results prompt further investigation into possible emergence of new geographical distribution of WNV in northern Europe.

Large herbivores are known as tick mating and feeding sites [33] and could be important in amplifying TBEV vector populations [34], therefore bigger stables could possibly be a reason for increased tick numbers in the area. On the other hand, *Ixodes* tick density do not necessarily correlate with TBEV spread in susceptible species and horses may be held responsible for diverting tick bites from competent hosts, thus diluting pathogen transmission [35]. Pastures and riding trails of horses can theoretically be a suitable medium for TBEV foci as several papers denote the effect of grazing as well as influence of litter layer thickness on tick population [36,37], but continuous multimodal works are required to accurately evaluate the role of horses in the natural spread of the virus.

## 5. Conclusions

Horses are dead-end hosts of TBEV providing measurable immunity responses upon natural infection with TBEV that seem to accurately represent infectivity of an area. TBEV surveillance studies in horses can reveal new microfoci and permit epidemiological mapping on lower than district level, therefore equines can be attributed as possible TBEV sentinel species.

## Figures and Tables

**Table 1:** Results of equine serology and PCR of serum and ticks in 32 tested stables.

No. of the Stable	Sample Size of the Stable	County	Seroprevalence in the County	TBEV Seropositive Horses	Seroprevalence in the Stables	CI 95 %	PCR Positive Serum Samples	Tick Pools	PCR Positive Tick Pools
1	12	Klaipėda	42.3	8	66.6	34.8–90.0	0	4	0
2	14			3	21.4	0.4–50.8	0	2	0
3	12	Telsiai	8.3	1	8.3	0.2–38.4	0	0	0
4	7	Siauliai	30.6	4	57.1	18.4–90.1	1	1	0
5	10			1	10.0	0.2–44.5	3	0	0
6	19			6	31.5	12.5–56.5	0	0	0
7	12	Panevezys	46.2	5	41.6	15.1–72.3	1	0	0
8	14			7	51.0	23.0–76.9	1	0	0
9	13	Utena	30.8	4	30.7	0.9–61.4	0	0	0
10	3			1	33.3	0.8–90.5	0	0	0
11	4			1	25.0	0.6–80.5	0	0	0
12	11			4	36.3	10.9–69.2	0	3	0
13	15			5	33.3	11.8–61.6	1	2	0
14	6			1	16.6	0.4–64.1	0	0	0
15	8	Vilnius	50.6	1	12.5	0.3–52.6	0	1	0
16	6			6	100.0	54.0–100	0	1	0
17	2			1	50.0	0.1–98.7	0	0	0
18	14			14	100.0	0.7–100	0	22	4
19	17			9	52.9	27.8–77.0	0	0	0
20	17			2	11.7	0.1–36.4	2	0	0
21	17			7	41.1	18.4–67.0	0	0	0
22	6			4	66.6	22.2–95.6	0	0	0
23	2	Marijampole	22.2	2	100.0	15.8–100.0	0	0	0
24	7			0	0.0	0–40.9	0	0	0
25	4	Kaunas	34.2	4	100.0	39.7–100.0	0	1	0
26	6			2	33.3	0.4–77.7	0	0	0
27	8			2	25.0	3.1–65.0	0	0	0
28	13			5	38.4	13.8–68.4	0	0	0
29	1			0	0.0	0.0–97.5	0	0	0
30	6			0	0.0	0.0–45.9	0	0	0
31	5	Alytus	20.0	2	40.0	0.5–85.3	0	0	0
32	10			1	10.0	0.2–44.5	3	1	0
Total	301			113	37.5	32.3–43.1	12	38	4

## Data Availability

The data presented in this study are available on request from the corresponding author. The data are not publicly available due to confidentiality agreements.

## Supplementary Materials

The following are available online at https://www.mdpi.com/2076-0817/10/2/140/s1, Figure S1: Standard curve of qPCR using serial dilutions of stock DNA.
