# Supplementary figures and images for: Cross-Sectional Study on the Prevalence and Factors Influencing Occurrence of Tick-Borne Encephalitis in Horses in Lithuania
Source: Pathogens. 2021 Jan 31;10(2):140. doi: 10.3390/pathogens10020140 (PMC7911650; doi:10.3390/pathogens10020140)

## Supplementary material

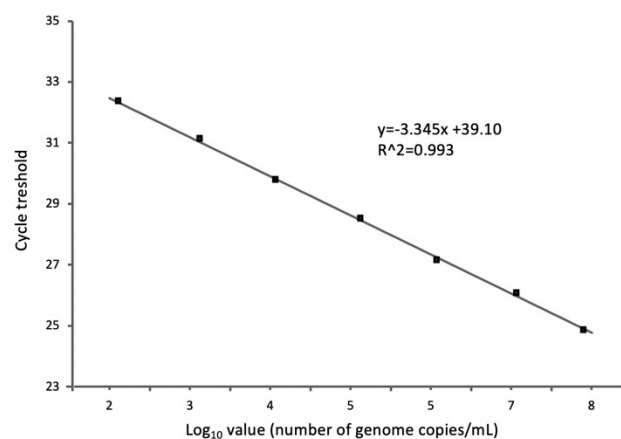

Figure S1. Standard curve of qPCR using serial dilutions of stock DNA.

Supplement: Supplementary file 1 [file pathogens-10-00140-s001.pdf]
